# Optimizing Care: Integrative Oncology in Myeloproliferative Neoplasm

**DOI:** 10.1007/s11912-024-01568-9

**Published:** 2024-07-05

**Authors:** Shagun Singh, Supriya Peshin, Ashley Larsen, Krisstina Gowin

**Affiliations:** 1 Internal Medicine, Banner University Medical Center, Tucson, AZ USA; 2 Norton Community Hospital, Ballad Health, Norton, VA USA; 3 Department of Medicine, University of Arizona, Tucson, AZ USA; 4 Department of Medicine, Hematology Oncology, University of Arizona, 1501 N Campbell Ave, Tucson, AZ 85724 USA

**Keywords:** Myeloproliferative neoplasm, Integrative oncology, Symptom burden, Mindfulness, Yoga, JAK inhibition

## Abstract

**Purpose of Review:**

Myeloproliferative neoplasm (MPN) burdens the lives of those affected. MPN patients endure significant impacts on their physical, psychological, and social well-being. While pharmacological interventions offer some disease and symptom control, they often have unfavorable side effects. This review explores the potential of Integrative Oncology (IO) therapies in managing MPNs and their associated symptoms.

**Recent Findings:**

IO is dedicated to augmenting conventional treatments through integrating interventions targeting the mind, body, nutrition, supplements, and other supportive care therapies. Several small studies suggest the benefit of an IO approach in MPN patients. These benefits are postulated to be modulated through enhanced physical capacity, reduced disease-related inflammation, subconscious mind training, and gut microbiome modulation.

**Summary:**

By combining IO with evidence-based pharmacological treatments, the potential exists to enhance the quality of life and clinical outcomes for individuals with MPNs. Future research should prioritize well-powered studies, including diverse demographics and symptom profiles, with appropriate study duration, to draw definite conclusions regarding the observed effects.

## Introduction

Myeloproliferative neoplasms (MPN) represent a complex disorder of hematopoietic stem cells characterized by clonal expansion of mature myeloid cells [1]. The revised WHO and International consensus criteria recognize three main subgroups: Polycythemia Vera (PV), Essential Thrombocythemia (ET), and Primary Myelofibrosis (PMF) [2, 3]. Despite their relatively low global annual incidence rates (PV at 0.84 cases, ET at 1.03, and PMF at 0.47/100,000), patients with MPNs face the chronic course of the disease, with survival measured in decades [4]. This chronicity brings forth a myriad of challenges, including increased risk of thrombosis-associated cardiovascular events, microvascular symptoms, splenomegaly, potential for leukemic transformation, and various constitutional symptoms [5]. Consequently, patients endure years of physical discomfort, cognitive impairment, diminished quality of life, and decreased work productivity [6, 7].

Our understanding of the pathological mechanisms underlying MPNs continues to evolve. Common somatic diseases driver mutations, such as Janus Kinase2 (JAK2), Calreticulin (CALR), and mutations in the thrombopoietin receptor (MPL), drive signal transduction and transcription activation. This is often accompanied by inflammatory cascades involving interleukins IL-1, IL-4, IL-6, IL-8, and tumor necrosis factor (TNF) [8, 9]. Extrinsic risk factors like older age, male sex, increased body mass index (BMI), smoking, gut microbiome dysbiogenesis, ionizing radiation, benzene exposure, and low socio-economic status are linked with disease onset and progression [10].

Standard therapies for MPN include phlebotomy, aspirin, cytoreduction, interferons, JAK-inhibition, allogeneic stem cell transplant (for MF), and many new therapeutics in the pipeline [11]. While treatment aims to reduce symptom burden and improve survival, there are unmet challenges with chronic use. These include residual MPN symptoms not ameliorated with pharmacological therapy or side effects from treatments themselves, such as fatigue with phlebotomy, bleeding tendencies with aspirin, increased inflammation with interferons, dose-dependent anemia, and thrombocytopenia with JAK inhibition, and risks of secondary cancers [12–15]. The second-line JAK inhibitors like fedratinib, pacritinib, and momelotinib show promise to cytopenic patients, yet are associated with other potential side effects [12]. Modulating MPN and associated symptoms is complex, with multiple inflammatory and non-inflammatory pathways playing a role [16]. Treatment response can vary significantly due to the heterogeneous clinical and phenotypical features of MPNs. Therefore, symptom management relies on comprehensive tools like the Myeloproliferative Neoplasm Symptom Assessment Form- Total Symptom Score (MPNSAF-TSS) to gauge treatment efficacy [17].

Despite therapeutic advancements, treatment-related side effects and inconsistent responses persist, prompting interest in an Integrative Oncology (IO) approach. IO is defined as a patient-centered, evidence informed filed of cancer care that utilizes mind and body practices, natural products, and/or lifestyle modifications from different traditions alongside conventional cancer treatments. IO aims to optimize health, quality of life, and clinical outcomes across the cancer care continuum and to empower people to prevent cancer and become active participants before, during, and beyond cancer treatment. This encompasses a range of modalities, including exercise, mind–body therapies like yoga, meditation, tai-chi, and qigong, as well as dietary interventions and supplements [18••]. There is an increased utilization of these minimal-risk interventions among cancer patients. These interventions empower patients to actively manage their health, mitigate treatment-related complications, and potentially augment the effectiveness of cancer treatment [19–23]. This review will explore the available literature on the efficacy, feasibility, dosage-frequency recommendations, adverse effects or contraindications, and perceptions of IO in alleviating the symptom burden associated with MPN. (Table 1).

**Table 1.**
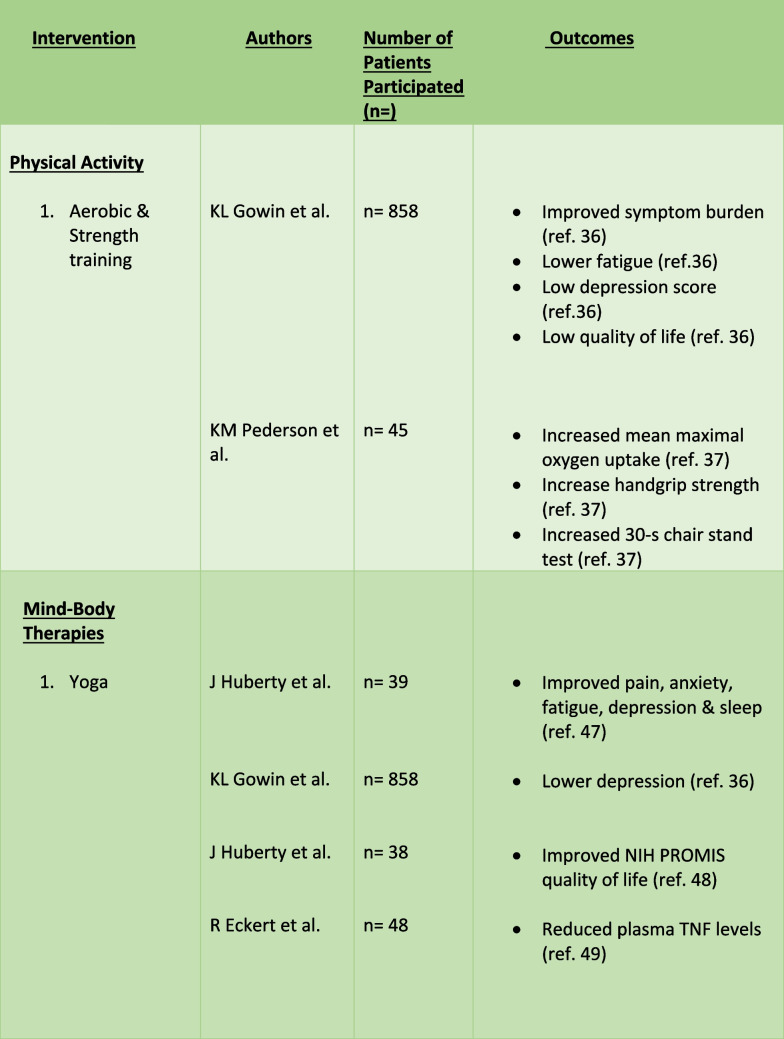

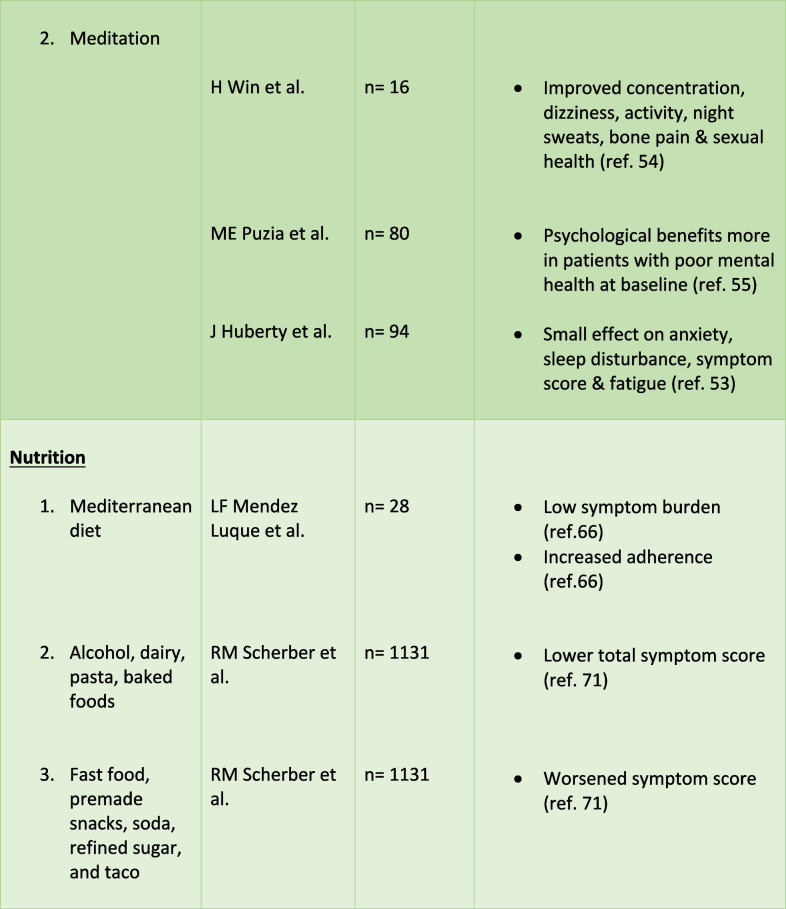

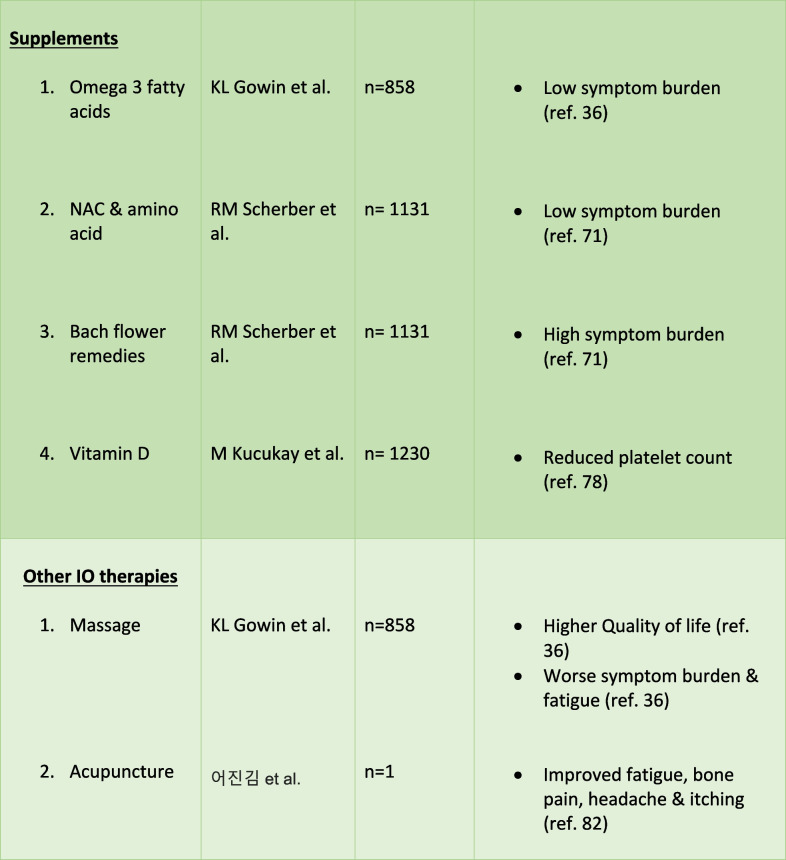
Outcomes of IO in MPN

## Physical activity (PA)

Most research on targeted exercise interventions in hematological malignancies centers around leukemia, lymphoma, and multiple myeloma; however, emerging evidence suggests positive outcomes in MPNs [23]. Exercise yields significant improvement in cancer treatment-related adverse effects, quality of life, psychosocial distress, fatigue, cognitive function, and sexual health in cancer patients [24]. The physically active population is at a lower risk of some cancers, such as esophageal, liver, colorectal, lung, leukemia, and melanoma [25]. Exercise is further associated with low all-cause and cancer-specific mortality in breast and colorectal cancer [26]. One potential explanation of exercise benefit lies in the upregulation of immune pathways, as exercise-induced mobilization of natural killer (NK) cells and release of IL-6 reduced tumor size in murine models [27–29]. Exercise may also impact insulin-related growth-promoting biomarkers, as observed in breast cancer [30]. Additionally, enhanced physical tolerance to cancer treatment, resulting in increased completion rates, and observed favorable epigenetic modifications are among the mechanisms through which exercise confers benefit [31, 32].

Specific to the MPN population, where the risk of vascular thrombosis is notably elevated, exercise can be instrumental in attaining cardiovascular fitness by boosting fibrinolytic activation [33]. MPN patients frequently report more significant fatigue and lower quality of life associated with a sedentary lifestyle [34, 35]. One large multicenter international survey based analysis observed lower symptom burden (p = 0.01) and depression (p = 0.006) with aerobic exercise and strength training. Another small-scale preliminary study noted similar results with no significant improvement in fatigue and quality of life following a 12-week exercise program [37]. These discrepancies may be attributed to various factors, including differences in patient characteristics, adherence to exercise programs, and study methodologies [36•, 37]. Nevertheless, evidence indicates improved upper and lower body strength (p = 0.01, p < 0.001) and increased maximal oxygen uptake (p = 0.01) with physical intervention in MPN patients, suggesting the feasibility of PA in this population [37].

Questions regarding the optimal frequency, intensity, and type of exercise in MPNs remain unanswered. Proposed recommendations are based on NCCN guidelines for physical activity in cancer survivors, advocating for at least 150 min per week of combined moderate-intensity aerobic and strength training exercises performed at least two or more times a week [38]. Patients with PV were observed to prefer individualized training regimes, mainly conducted outdoors, averaging 45–60 min per week, at least twice a week [39]. Patients with specific risks like splenomegaly, balance issues, bleeding tendencies, and skin symptoms could have modified training like avoiding contact or ball sports, low injury training, and Ultraviolet light protection while outdoors. Identified barriers to PA include anxiety, depression, and fear of injuries from maximal intensity exercise. Therefore, incorporating cognitive therapies and tailoring training programs to individual needs can complement the effects of PA and may help overcome these barriers [39, 40].

## Mind–body Therapies

Mind–body therapies (MBT) combine a delicate synergy of mental focus, physical postures, and breathing exercises that contribute to overall well-being [41]. They may include yoga, meditation/mindfulness, tai-chi, and qigong. MBT has demonstrated positive effects on various aspects of cancer-related distress, including anxiety, depression, sleep disturbances, and pain [42•]. The underlying mechanisms of these benefits are intricate, likely involving a reduction in inflammation (observed via reduced inflammatory markers like C reactive protein (CRP) and IL-6) and alteration in leukocyte gene transcriptions such as downregulation in pro-inflammatory NF-kB genes and activation of glucocorticoid receptor gene [43]. Observed effects may also stem from the modulation of autonomic nervous and central nervous system pathways. Increased mindfulness, decreased rumination, and enhanced self-compassion mediate beneficial effects derived from mindfulness practices within MBT [44].

Studies have shown that yoga is associated with enhanced psychosocial outcomes in other cancer types, such as breast cancer and lymphoma patients [45]. Similarly, positive results, including improved sleep, symptom scores, pain, anxiety, and depression, have been observed in MPN patients who engaged in 60 min of online yoga weekly for 12 weeks [46]. MPN patients linked yoga with improved circulation, eating habits, breathing, and an increased sense of enjoyment [46, 47]. The feasibility of yoga in MPN is supported by high adherence to the regime, satisfaction with outcomes, and follow-up rates exceeding 70% in yoga studies [48].

Additionally, decreased plasma TNF level was seen with yoga in MPN, and no effect on IL-6 was seen [49]. Notably, the beneficial effect of yoga was more pronounced in overweight MPN patients [50]. Adverse events such as spleen irritation have been reported but can be mitigated with pose modifications [46, 49]. Similar to PA, barriers to engaging in yoga therapies for cancer patients can include fatigue, pain, and transportation issues. These obstacles can be lessened by opting for remote participation and starting with minimal therapy doses [51]. The studies did not provide specific recommendations regarding type, dose, and frequency of yoga. Mild to moderate intensity Hatha and Vinyasa style yoga was included, and interestingly, yoga exceeding 60 min a week did not confer additional benefits. Therefore, large randomized control trials (RCT) are needed to explore this aspect [46, 49].

Mindfulness, the deliberate practice of focusing on the present moment without judgment, offers a pathway to cultivating control and wisdom in one’s life [52]. In the realm of MPNs, digital mindfulness applications like Calm and My Wellness Coach (MWC) have garnered attention for their feasibility and efficacy. Users reported satisfaction with these apps, often recommending them to fellow patients [53, 54]. There is evidence that these apps alleviate anxiety and depression, pervasive issues within the MPN community, particularly benefitting those grappling with existing mental health challenges [55]. MWC, with its emphasis on goal setting and behavior modification, demonstrated positive impacts on various symptoms, including impaired concentration, dizziness, night sweats, bone pain, sexual health and sedentary behavior [54]. Patients noted a profound sense of calmness and improved well-being while engaging in these platforms [56]. However, the anticipated improvement in sleep disturbances was not as pronounced, possibly due to relatively short trial durations (4–8 weeks in Calm and 12 weeks in MWC). While the accessibility of these apps via online platforms was appreciated, challenges such as internet connectivity issues, intrusive reminders, and technology barriers among older individuals were noted [54, 56]. Further investigation is warranted to determine the optimal dosage and type; a study found no discernible variance in mental wellness scores between individuals practicing 10 versus 30 min of mindfulness [57].

## Nutrition

The World Cancer Research Fund (WCRF) and its affiliates, such as the American Institute for Cancer Research (AICR), champion dietary regimens aimed at cancer prevention and supporting cancer survivorship [58••]. Within the spectrum of cancer care, **d**iet and weight management play an integral role, as underscored by the ABC**D**Es of cancer care according to NCCN guidelines [59]. Nutrition interventions have long been linked with better cancer outcomes and enhanced cardiovascular health through their anti-inflammatory and antioxidant properties [60]. Notably, individuals with MPN face a heightened risk of cardiovascular issues from increased inflammation, defective macrophage lipid efflux, release of reactive oxygen species, neutrophil extracellular trap formation, and altered platelet function and form [61]. Interestingly, clonal hematopoiesis of indeterminate potential (CHIP), often a precursor to MPN, also independently correlates with atherosclerosis, highlighting the importance of early dietary interventions [62]. While tailored dietary guidelines for MPNs continue to evolve, recommendations from the WCRF and AICR generally advocate for a daily intake of 30 g of fiber alongside a variety of whole grains, pulses, and five servings of non-starchy vegetables and fruits for cancer patients [58••].

The Mediterranean diet, particularly when supplemented with extra virgin olive oil (EVOO), has shown promise in reducing major cardiovascular events in high-risk populations due to its anti-inflammatory properties of phenolic compounds in EVOO and potential role in gut microbiome modulation, which is pivotal in innate immune system development and hematopoiesis regulation [63–65]. The Mediterranean diet was found to be safe and had a high Mediterranean Diet Adherence Score (MEDAS > 8) compared to the conventional U.S. Dietary Guidelines Americans (USDA) diet among MPN patients. The Mediterranean diet was also associated with low symptom burden in MPNs in a small study [66]. Additionally, a low level of low-density lipoprotein ( -13 g/dl) was observed with nine weeks of the Mediterranean diet, prompting a discussion on its potential role in mitigating cardiovascular morbidity in individuals with MPNs. However, sustained adherence and ongoing education are essential for maintaining these positive outcomes [66]. Conversely, no change in inflammatory markers, gut microbiome composition, and JAK allele burden was observed with the Mediterranean diet in MPNs. This can be attributed to shorter duration of intervention, differences in diet strength, and less diverse gut microbiome in industrialized countries [66, 67].

Other dietary factors, such as coffee, should be considered. Interestingly, an inverse relationship between coffee consumption and PV risk was reported, possibly attributed to the anti-inflammatory and antitumor properties of bioactive compounds present in coffee [68–70].

MPNs consuming alcohol (p = 0.001), dairy products (p = 0.02), pasta (p = 0.02), and baked foods (p = 0.02) reported lower total symptom scores (MPNSAF-TSS). However, fast foods (p = 0.07), pre-made snacks (p = 0.03), soda (p < 0.0001), refined sugar (p = 0.01), and tacos (p = 0.03) observed worse MPNSAF-TSS [71]. Similar observations associated increased risk of PV with sugar consumption from fruits like citrus, melon, and berry [72]. While the associations between food intake and TSS are interesting, they are interpreted with caution given data was retrospective and patient-reported and thus, potentially biased and inaccurate. As such, more prospective and randomized studies are needed to explore this further. Barriers to implementing dietary changes include minimal data, food restrictions and intolerances due to disease, and logistical challenges like time and energy required for meal preparation [73].

## Supplements

Individuals with MPNs often turn to supplements to bolster their overall health, address nutritional deficiencies, and alleviate disease symptoms [36•, 71]. Research indicates that supplement usage is more prevalent among females, older, and physically active MPN patients [71]. Commonly consumed supplements among MPN patients include amino acid supplements, N acetyl Cysteine (NAC), bach flowers remedies, vitamin D, multivitamins, omega 3 fatty acids, calcium, turmeric, green tea, vitamin E, medical marijuana, and medicinal mushrooms [36•, 71]. While omega 3 fatty acids (p = 0.03), amino acid (p = 0.02), and NAC (p = 0.02) have been linked to lower symptom burden, consumers of bach flower remedies reported high symptom burden [36•, 71]. However, the remaining supplements showed no significant correlation with symptom burden, depression, fatigue, and quality of life in MPN patients [36•].

Omega fatty acids are polyunsaturated acids known for their anticancer properties, primarily attributed to their affinity for the cyclooxygenase pathway, which hampers the proliferation of helper T cells associated with cancer susceptibility and their anti-inflammatory effects [74].

NAC, through its anti-oxidant properties, inhibited thrombosis in JAK2V617F murine models and prevented in vitro extracellular trap formation in neutrophils from human MPNs [75]. Given that neutrophil extracellular trap formation exacerbates thrombosis in MPNs [61] and MPN cells exhibit resistance to oxidative stress, NAC holds promise as a therapeutic agent.

Moreover, vitamin D appears to influence macrophage differentiation via the vitamin D-linked receptor pathway, potentially implicating its role in macrophage-mediated myelofibrosis in JAK2 transgenic mice [76]. While vitamin D deficiency is prevalent among MPNs, its therapeutic implications remain ambiguous [77]. However, treatment with vitamin D demonstrated a significant reduction in platelet counts in vitamin D-deficient patients, which theoretically could be beneficial in managing vitamin D-deficient ET by decreasing elevated platelet levels [78].

Curcumin, a natural compound, has been found to have anti-proliferative and pro-apoptotic effects on JAK2V617F mutated MPN cells by inhibiting JAK2/STAT and mTOR1 signaling pathways [79]. Additionally, curcumin also negatively regulates JAK-STAT signaling by activating suppressors of cytokine signaling (SOCS 2 and SOCS3) and inhibiting histone deacetylase (HDAC1 and HDAC8), indicating its potential as a therapeutic strategy for JAK-positive MPNs [80].

While the evidence regarding supplement mechanism and impacts on MPNs are growing, more data is needed to recommend any supplementation in MPN patients. Patients should be encouraged to disclose and discuss supplementation with health care teams to avoid potential harm and drug interactions.

## Additional Integrative Oncology Therapies

Cancer patients often face significant challenges in receiving clear information about IO from their treating team, leading many to seek support from alternative healthcare providers, such as chiropractors, naturopathic doctors, and acupuncturists [81]. This trend has resulted in a rise in the utilization of modalities such as massage, homeopathy, and acupuncture within the cancer community. In the context of MPN, research indicates an increased prevalence of complementary therapies, with statistics revealing the use of massage (28.4%), acupuncture (19.3%), chiropractic (16.2%), aroma therapy (8.6%), homeopathy (6.3%), reiki (5.8%), Ayurveda (2.8%), and hypnosis (2%) among MPN patients [36•].

Interestingly, MPN patients who incorporate massage into their care regimen report a higher quality of life (p = 0.04), albeit with heightened symptom burden and fatigue (p < 0.01); likely reflecting the differences in individual symptom profiles [36•]. Furthermore, a case report showed that thirteen acupuncture sessions over six months improved fatigue, bone pain, headache, and itching in a PV patient [82]. Massage observed benefits can be from neurotransmitter modulation, like increased serotonin and dopamine and decreased cortisol and norepinephrine levels [83]. Similarly, acupuncture’s efficacy is due to its influence on neurotransmitters, gene alteration, and opioid upregulation [84].

**Fig. 1:**
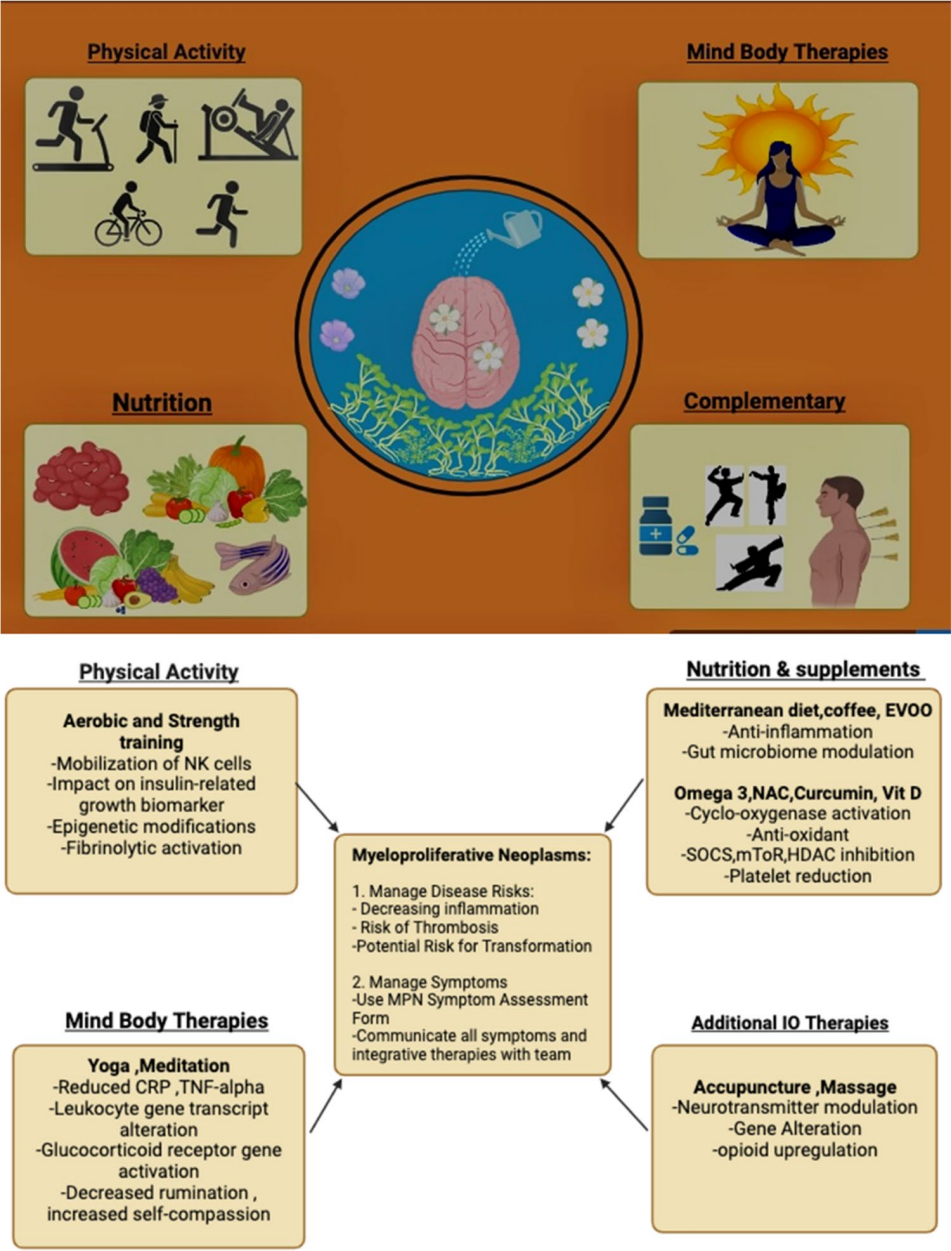
IO mechanisms and MPN risk management

Given the growing interest in these interventions, it is imperative that oncologists facilitate open communication with patients and guide them toward reputable integrative care resources. This proactive approach not only empowers patients to make informed decisions but also ensures their safety and well-being throughout their cancer journey. (Fig. 1).

## Conclusion

Individuals with MPN often protract a prolonged survival period. The armamentarium of pharmacological therapy continues to grow, offering promise in controlling the disease and ameliorating symptom burden, but patients continue to have unmet needs. Recent research indicates that IO can potentially enhance the physical, social, and psychological well-being of MPN patients, as well as potentially mitigate cardiovascular outcomes at minimal cost and low risk. Clinicians should integrate their clinical expertise in pharmacotherapy with evidence-based IO approaches to optimize patient care, considering patient’s preferences and realistic expectations. However, the current understanding of IO in MPN exhibits variability and represents a small sample size, preventing definite conclusions. Future studies should focus on larger, longer term studies with standardized interventions, control groups, and study methodologies limiting social desirability and recall bias. Additionally, investigations should also prioritize diverse participant cohorts while adjusting for modifiable risk factors, such as gender, BMI, and educational background, to establish more robust evidence in this domain.

## Data Availability

No datasets were generated or analysed during the current study.
